# Multi-Gene Next-Generation Sequencing for Molecular Diagnosis of Autosomal Recessive Congenital Ichthyosis: A Genotype-Phenotype Study of Four Italian Patients

**DOI:** 10.3390/diagnostics10120995

**Published:** 2020-11-24

**Authors:** Tiziana Fioretti, Luigi Auricchio, Angelo Piccirillo, Giuseppina Vitiello, Adelaide Ambrosio, Fabio Cattaneo, Rosario Ammendola, Gabriella Esposito

**Affiliations:** 1 CEINGE—Advanced Biotechnologies s.c. a r.l., Via Gaetano Salvatore, 80145 Naples, Italy; fioretti@ceinge.unina.it; 2 Department of Clinical Medicine and Surgery, University of Naples Federico II, Via S. Pansini, 5, 80131 Naples, Italy; luauricc@unina.it; 3 San Carlo Hospital, Operating Unit of Dermatology, 85100 Potenza, Italy; picciangelo@yahoo.it; 4 Complex Operating Unit of Medical Genetics, University Hospital Federico II, Via S. Pansini, 5, 80131 Naples, Italy; dr.giuseppina.vitiello@gmail.com; 5 Department of Molecular Medicine and Medical Biotechnologies, University of Naples Federico II, Via S. Pansini, 5, 80131 Naples, Italy; adelaideambrosio22@gmail.com (A.A.); fabio.cattaneo@unina.it (F.C.); rosario.ammendola@unina.it (R.A.)

**Keywords:** congenital ichthyosiform erythroderma, genotype-phenotype correlation, large deletion, nonsyndromic autosomal recessive ichthyosis, next generation sequencing

## Abstract

Autosomal recessive congenital ichthyoses (ARCI) are rare genodermatosis disorders characterized by phenotypic and genetic heterogeneity. At least fourteen genes so far have been related to ARCI; however, despite genetic heterogeneity, phenotypes associated with mutation of different ARCI genes may overlap, thereby making difficult their clinical and molecular classification. In addition, molecular tests for diagnosis of such an extremely rare heterogeneous inherited disease are not easily available in clinical settings. In the attempt of identifying the genetic cause of the disease in four Italian patients with ARCI, we performed next-generation sequencing (NGS) analysis targeting 4811 genes that have been previously linked to human genetic diseases; we focused our analysis on the 13 known ARCI genes comprised in the panel. Nine different variants including three novel small nucleotide changes and two novel large deletions have been identified and validated in the *ABCA12*, *ALOX12B*, *CYP4F22*, and *SULT2B1* genes. Notably, two patients had variants in more than one gene. The identification and validation of new pathogenic *ABCA12*, *ALOX12B*, *CYP4F22*, and *SULT2B1* variants through multi-gene NGS in four cases of ARCI further highlight the importance of these genes in proper skin function and development.

## 1. Introduction

Autosomal recessive congenital ichthyoses (ARCI) are clinically and genetically heterogeneous nonsyndromic inherited disorders of keratinization with variable severity, characterized by skin scaling and hyperkeratosis of varying degree [1,2]. ARCI are very rare, with an estimated prevalence in Europe of about 1:100,000 [3]. ARCI newborns often present the collodion membrane, which may cause serious perinatal complications, leading in some cases to infant mortality [3,4]. Once the collodion membrane sheds, the underlying skin phenotype can range from harlequin ichthyosis (HI) to lamellar ichthyosis (LI), characterized by dark plate-like scales, and congenital ichthyosiform erythroderma (CIE), with fine whitish scaling and variable erythroderma [5]. In some cases, collodion shedding leaves minor or no skin impairment, as in the self-improving collodion ichthyosis (SICI) [2,6]. Among ARCI, HI represents the most severe phenotype and is often lethal; however, even less severe types of ARCI may negatively influence patients’ quality of life [7,8].

In an agreement with the phenotypic heterogeneity, biallelic pathogenic variants in 14 genes, namely *ABCA12* (OMIM 607800), *ALOX12B* (OMIM 603741), *ALOXE3* (OMIM 607206), *CERS3* (OMIM 615276), *CYP4F22* (OMIM 611495), *CSTA* (OMIM 184600), *LIPN* (OMIM 613924), *NIPAL4* (OMIM 609383), *PNPLA1* (OMIM 612121), *POMP* (OMIM 613386), *SLC27A4* (OMIM 604194), *ST14* (OMIM 606797), *SULT2B1* (OMIM 604125), and *TGM1* (OMIM 190195), account for about 85% of nonsyndromic ARCI cases [4]. These genes encode proteins that participate in the proper building and functioning of the skin barrier through various pathways involved in lipid metabolism or lipid transport within the stratum corneum, and in the formation of the cornified envelope by protein cross-linking and lipid attachment [3]. With the exception of the severe HI, which is mainly due to *ABCA12* mutations, LI, CIE, and SICI phenotypes may occur as a consequence of pathogenic variants in most of the known ARCI genes, thereby making difficult clinical and molecular classification of the different types of ARCI [1,2,3,4,5,6,9,10,11]. On these bases neonatologists, pediatricians, and dermatologists share clinical management of these patients, but geneticists and molecular biologists are crucial to identify the underlying molecular defects and for exhaustive genetic counseling [12,13].

Despite advances in molecular biology, genetic tests searching for the molecular cause of very rare inherited diseases are not easily available in clinical diagnostic laboratories. Therefore, ARCI patients often remain without a genetic diagnosis and thus they are not fully aware of their condition [14]. Molecular characterization improves patient care by providing useful data for diagnostic classification and clinical management, genetic counseling, and prenatal diagnosis [9,15]. It also provides the basis for studies that aim to address the phenotypic and genetic complexity of different forms of a disease, but also for the design of new possible targeted therapies [3,16]. For all these reasons, it is important to define a successful molecular diagnostic strategy that is rapid, feasible and relatively inexpensive so that it can be applied to patients with ARCI and potentially to all patients suffering from similarly rare inherited diseases. The evolution of next-generation sequencing (NGS) technologies has changed the landscape of genetic testing, especially in rare inherited diseases, fostering its adoption in clinical settings. In particular, panel-based NGS assays are designed to reveal causal mutations in groups of genes associated with one or more genetic diseases [14]. Indeed, this technology can be successfully applied to molecular diagnosis of diseases with genetic heterogeneity, including allelic and locus heterogeneity, overlapping phenotypes, and with causal genes involved in common disease-related pathways [14,17]. Broadening our vision, a panel-based NGS assay that includes most genes implicated in human diseases can be useful in clinical settings to analyze any patient affected from a known condition associated with mutations in one or multiple known genes [18,19,20].

Herein, we report molecular characterization of four ARCI patients who have been waiting for a molecular diagnosis for over 20 years. We applied NGS-based analysis targeting a panel of 4811 genes implicated in human diseases, which included most of the genes hitherto related to Mendelian disorders. Data analysis focused on the ichthyosis-related genes revealed pathogenic or likely pathogenic sequence variants in all the analyzed patients and gave hints to look beyond the mere NGS data, thereby revealing novel disease-alleles and unexpected genotypes to be related to the patients’ ichthyosis phenotypes.

## 2. Materials and Methods

### 2.1. Study Subjects and Ethical Approval

Four unrelated Italian patients, two females and two males, had a clinical diagnosis of ARCI, as assessed by a dermatologist. All the patients were born encased in a collodion membrane. Family histories were negative for inherited diseases or any skin disorder, except one family that had a previous newborn suffering with a lethal form of collodion baby. One patient had consanguineous parents. Healthy parents or relatives were recruited, when available. All the participants gave written informed consent before undergoing the molecular analysis, which was performed for clinical diagnostic purposes. The study was approved by the Ethical Committee of University Federico II, Naples, Italy (protocol n. 370/18, approval date: 30/01/2019), and carried out in accordance with the Declaration of Helsinki.

### 2.2. Genomic DNA Extraction and Targeted NGS

Genomic DNA was extracted from peripheral blood leukocytes with a QIAamp DNA Mini Kit (QIAGEN Italia, Milan, Italy). Library preparation was carried out according to the manufacturer’s instructions with a TruSight One Sequencing Panel (v.1.1) (Illumina, San Diego, CA, USA), a prepackaged panel-based NGS assay targeting the coding exons of 4811 genes, most of them associated with known Mendelian diseases. Library sequencing was performed with a NextSeq 500 sequencing system using a High Output PE 300 Cycles flow-cell (Illumina, San Diego, CA, USA).

### 2.3. NGS Data Processing and Classification of Variants

Pipeline for NGS data analysis was limited to the ichthyosis-related genes. Reads were aligned against the reference genome (GRCh37/hg19). Genome Analysis Toolkit (GATK) and ANNOVAR [21] were used to call, annotate, filter, and prioritize variants. Integrative Genomics Viewer (IGV) software was used to display NGS data (read depth and coverage). For classification of small nucleotide variants (SNVs), we interrogated the Genome Aggregation Database (gnomAD; https://gnomad.broadinstitute.org/), the Single-Nucleotide Polymorphisms Database (dbSNP; https://www.ncbi.nlm.nih.gov/projects/SNP/), HGMD Professional (https://www.biobase-international.com/product/hgmd), PubMed (https://www.ncbi.nlm.nih.gov/pubmed/).

We focused on SNVs with minor allele frequency (MAF) of ≤0.002, as reported in the consulted databases. To evaluate pathogenicity of SNVs, we interrogated the human genomic variant search engine Varsome and the following bioinformatic tools: Mutation Taster (https://www.mutationtaster.org/), PolyPhen-2 (https://genetics.bwh.harvard.edu/pph2/), SIFT (https://sift.jcvi.org/), Provean (https://provean.jcvi.org/index.php), Human Splicing Finder version 3.0 (https://www.umd.be/HSF3/), NetGene2 (https://www.cbs.dtu.dk/services/NetGene2/), Alamut version 0.9 (https://www.fruitfly.org/).

To detect copy number variants (CNVs), relative quantification of genomic DNA sequences was carried out by calculating the ratio between the read depth reported for each exon of the gene of interest (GOI), i.e., *ABCA12* (NM_173076.3), *ALOX12B* (NM_001139.3), *CYP4F22* (NM_173483.4), and *SULT2B1* (NM_177973.2), and the average read depth of all the exons of a reference autosomal gene (*AGRN*) in patients, relative to the average ratio between the read depth of each target exon normalized to the reference gene observed in three controls. Relative gene dosage was expressed as fold change (two copies = 0.85–1.20; one copy = 0.35–0.65).

### 2.4. Variants’ Validation

To confirm putative pathogenic SNVs, DNA fragments corresponding to *ABCA12, ALOX12B* and *CYP4F22* mutated exons were amplified by polymerase chain reaction (PCR) and analyzed by Sanger sequencing. Assembly of DNA sequences was obtained by the software package AutoAssembler (Applied Bio-systems, Foster City, CA, USA). Deletions were confirmed by quantitative real-time PCR (qPCR) performed in triplicate on genomic DNA samples using an iCycler™ version 3.021 (Bio-Rad Laboratories S.r.l., Segrate, Italy) with opportune primer pairs (Table 1) and a iQ^TM^SYBR^®^Green Supermix (Bio-Rad Laboratories S.r.l., Segrate, Italy). Fluorescent signal intensity was recorded and analyzed by iCycler™ iQ Optical System software v3.0a (Bio-Rad Laboratories S.r.l., Segrate, Italy). The human *HBB* gene was chosen as an autosomal reference gene. For each GOI, the data from probands were expressed as fold change relative to data from three normal subjects, using the comparative ΔC_T_ method [22].

Independent segregation of the identified variants was demonstrated in three cases. Variants were annotated according to HGVS recommendations [23] and interpreted according to American College of Medical Genetics and Genomics (ACMG) guidelines [24].

## 3. Results

### 3.1. Clinical and Molecular Analysis

To identify the genetic cause of ARCI in four Italian patients, we used a multi-gene NGS assay targeting most genes related to Mendelian disorders, including 13 of the 14 genes hitherto associated with ARCI. In the analyzed patients, this approach identified putative disease-causing variants in five genes previously associated with ARCI (Table 2).

#### 3.1.1. Affected Individual 1

**Figure 1:**
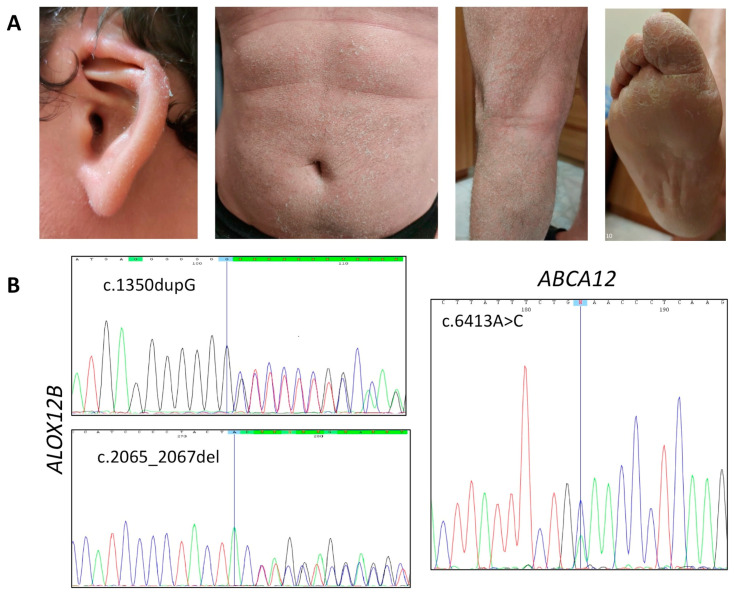
Phenotypic and genetic features of patient 1. (**A**) Overfolded ear, patchy erythema associated with white scales, which are largest and grey on the legs, and plantar hyperkeratosis. (**B**) Sanger sequencing electropherograms showing the *ALOX12B* small deletions at the heterozygous state (**left**), and the heterozygous *ABCA12* nucleotide change leading to the missense variant p.Glu2138Ala (**right**).

Patient 1 is currently a 32-years-old male who was born preterm at 35 gestation weeks as collodion baby; notably, collodion was lethal for a previous child in this family. Since infancy, the patient showed white scaling on the whole body, with patchy erythema under the desquamated skin. Hyperkeratosis affected his legs and arms, which were characterized by larger and gray scales; plantar hyperkeratosis was also evident, with palmar hyperlinearity. He also had moderate scalp involvement without hypotrichosis, deformed auricle, and diffused facial erythema (Figure 1A).

The patient also experienced anhidrosis resulting in serious discomfort caused by a widespread burning sensation associated with erythema, which sometimes led him to fainting. He also acquainted transient hearing loss due to skin scales that built up in the ear canal, and dry eyes due to tear canal filling. He was in treatment with topical hydrating and keratolytic agents.

NGS revealed heterozygous SNVs in two ARCI-related genes, namely *ABCA12* and *ALOX12B*. Variants located within *ABCA12* were the known c.7093G>A (p.Asp2365Asn; rs726070) and the novel c.6413A>C (p.Glu2138Ala); they segregated independently in the patient’s family, as assessed by Sanger sequencing (Figure 1B). The first was classified as likely benign variant, since it resulted far more frequently (MAF = 0.028) in the general population than it would be expected, given the disease’s rarity. The variant c.6413A>C, not reported in public databases, changed the charged Glu2138, located in a loop between two transmembrane domains of the ABCA12 transporter, with a neutral Ala residue, and met the supporting ACMG criteria PM2 and PP3. Varsome classified this variant as variant of uncertain significance (VUS). On these bases, we concluded that mutation of *ABCA12* was not the main cause of ARCI in this family.

Consistently, NGS detected two SNVs within *ALOX12B* that we considered the actual cause of the disease. Indeed, a novel pathogenic frameshift variant c.1350dupG (p.Leu451Alafs*), likely leading to protein truncation, and the known in-frame deletion c.2065_2067del (p.Tyr687del; rs1414018786) were identified and confirmed by Sanger sequencing (Figure 1B).

The latter variant was extremely rare (MAF = 0.000008) and resulted in loss of Tyr687 located within the lipoxygenase catalytic domain, a highly conserved residue among different species, as determined by BLASTP analysis. Such amino acid loss may impair enzyme activity by altering the catalytic mechanism and/or protein folding [25]. The c.2065_2067del variant had never been associated before with ARCI and, therefore, Varsome classified it as a VUS, despite it meeting three ACMG pathogenicity criteria (PM1, PM2, PP3). As the two *ALOX12B* variants c.1350dupG and c.2065_2067del segregated independently in the patient’s family, in the overall evaluation of the effect of the c.2065_2067del (p.Tyr687del) variant on gene expression, we considered the ACMG pathogenicity criterion PM3 for recessive diseases, thereby concluding that it was a likely pathogenic variant. Thus, we considered the compound heterozygous genotype *ALOX12B*:c.[1350dupG];[2065_2067del] consistent with the patient’s phenotype.

#### 3.1.2. Affected Individual 2

**Figure 2:**
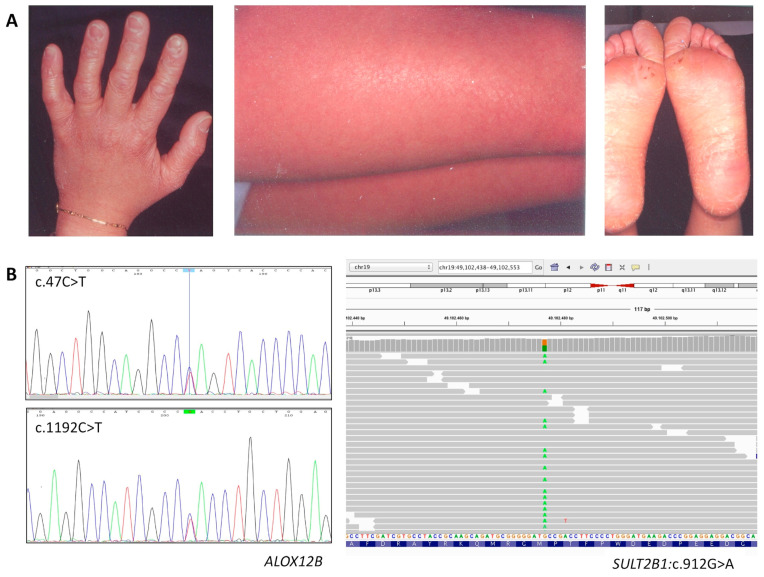
Phenotypic and genetic features of patient 2. (**A**) Marked palmoplantar keratosis and fine scaling with diffused erythroderma on the leg are shown. (**B**) Sanger sequencing electropherograms showing the nucleotide changes leading to the missense variants p.Ser16Leu and p.His398Tyr in *ALOX12B* (**left**); Interactive Genomics Viewer (IGV) detail of the variant revealing heterozygosity of the *SULT2B1* c.912G>A (p.Met304Ile) sequence change (**right**); the read depth for this nucleotide position is 162X, with the variant A allele sequenced 80X.

Patient 2 is currently a 40-years-old female who was born as collodion baby. In infancy she had fine white scaling on the whole body, with mild erythema under the desquamated skin. In adulthood, only very mild keratosis affected the legs and arms, whereas palmoplantar hyperkeratosis was evident (Figure 2A). She had no scalp involvement. Mild face erythema appeared only when she suffered from heat due to mild hypohidrosis; however, she suffered from frequent itching. She made daily use of topical moisturizers and emollients on the body.

NGS revealed two known heterozygous SNVs in *ALOX12B*, the c.47C>T (p.Ser16Leu; rs147784568) and the c.1192C>T (p.His398Tyr; rs752176414); both were very rare with MAF = 0.000012 and 0.000016 respectively (Figure 2B). Unlike the c.1192C>T, which was already associated with ARCI [26], this was the first time that the missense variant c.47C>T was found in an affected patient. Varsome classified c.47C>T (p.Ser16Leu) as a VUS, despite it meeting three ACMG pathogenicity criteria (PM1, PM2, PP2).

Unfortunately, we could not perform segregation analysis to verify if the c.47C>T and the c.1192C>T variants were located on independent alleles. Nevertheless, we considered these variants were pathogenic and therefore responsible of ARCI in this patient. Interestingly, NGS identified a heterozygous variant in another known ARCI-associated gene, namely, *SULT2B1* (Figure 2B). Although this known variant was relatively rare with a MAF = 0.001, Varsome classified it as likely benign variant.

#### 3.1.3. Affected Individual 3

Patient 3 is currently a 35-years-old male who was born as collodion baby. Parents were first cousins. In infancy, he presented fine white scaling with mild erythema on the whole body. In adulthood, only very mild face erythema and palmar hyperlinearity were evident. He only used topical emollients and moisturizing gels daily.

**Figure 3:**
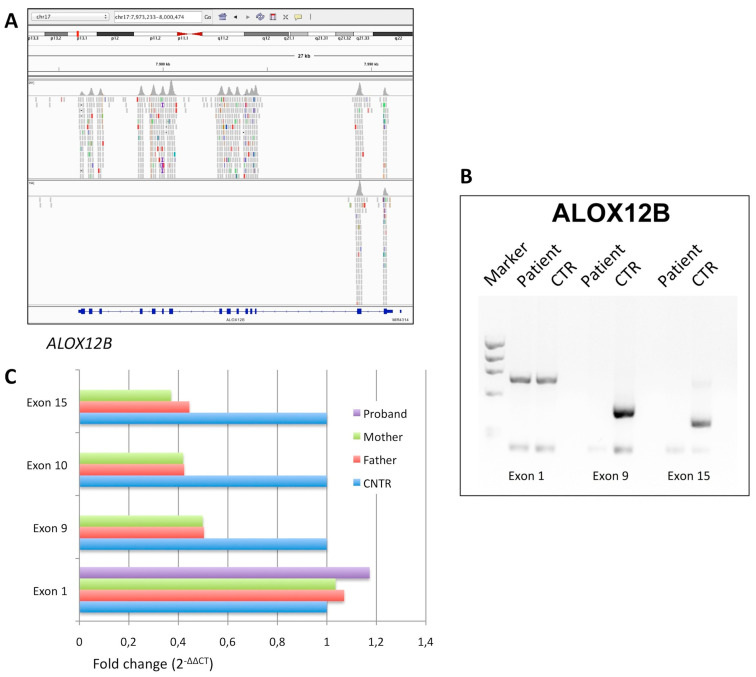
Genetic characterization of patient 3. (**A**) Integrative Genome Viewer detail of the whole *ALOX12B* gene locus in a control (top view) and in patient 3 (bottom view) revealed the homozygous loss of exon 3 to 15; the average read depth for *ALOX12B* coding region was about 70X in other patients analyzed. (**B**) Agarose gel electrophoresis analysis of the PCR products obtained for *ALOX12B* exon 1, 9, and 15, in patient 3 and in a normal control; in the corresponding pairs of lanes, the lower fragments represent an internal control of amplification conditions (see Materials and Methods). (**C**) Gene dosage by real-time qPCR analysis of *ALOX12B* exon 1, 9, 10, and 15, in patient 3 and in his heterozygous parents; signal quantification is normalized to an autosomal gene sequence and expressed as fold change with respect to a normal control, by using the 2^−ΔΔCt^ method.

NGS analysis did not reveal any disease-relevant SNV in the 13 ARCI genes captured in the panel. Therefore, we carried out an IGV visual analysis to evaluate read depth and coverage of all the ARCI gene-related exons. Surprisingly, we noted that exons from 3 to 15 of *ALOX12B* had no reads in patient 3 (Figure 3A), whereas they had an average read depth of about 70X (ranging from 30X to 100X) in the other patients analyzed in the same analytic session (Figure 3A). This finding indicated that patient 3 was homozygous for a large deletion removing genomic sequences including exon 3 to 15 of *ALOX12B*, as also confirmed by duplex PCR analysis (Figure 3B). In agreement, his consanguineous parents were heterozygous carriers of the deletion, as demonstrated by qPCR (Figure 3C).

#### 3.1.4. Affected Individual 4

**Figure 4:**
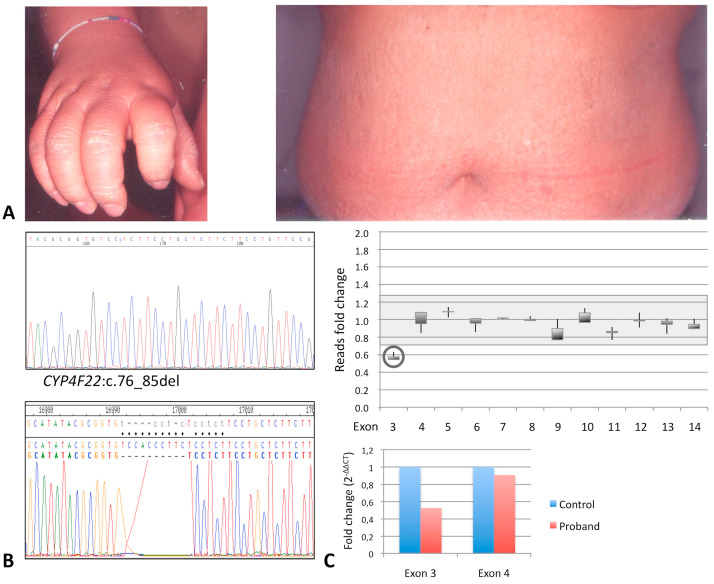
Phenotypic and genetic features of patient 4. (**A**) Marked hand hyperkeratosis and white-grey scaling with diffused erythroderma on the trunk are shown. (**B**) Screenshots of the sequence electropherogram (**top panel**) and of its graphical assembly with the normal gene sequence (**bottom panel**) show the ten-nucleotide deletion leading to the putative frameshift variant p.Thr26Serfs* in *CYP4F22*. (**C**) Gene dosage obtained in the patient by evaluating the read depth for each of the 12 *CYP4F22* coding exons (starting ATG within exon 3) normalized to the median read depth of all the exons of an autosomal gene (*AGRN*) (see Materials and Methods) and represented as fold change with respect to three normal controls (**top graph**). Real-time qPCR analysis of *CYP4F22* exon 3 in the proband; signal quantification is normalized to an autosomal gene sequence and represented as fold change with respect to a normal control (2^−ΔΔCt^ method) (**bottom graph**).

Patient 4 is currently a 43-years-old female who was born as collodion baby from nonconsanguineous parents. In infancy, she had large white-gray scaling on the whole body, with areas of erythema persistent in the adult age. Significant hyperkeratosis affected palmoplantar areas (Figure 4A), legs, and arms, which was more pronounced in elbows and knees. Trunk and back were covered with thin brownish scales; erythema was also present (Figure 4A). She had no scalp involvement, but she showed significant face erythema and hypohidrosis. She was in topical treatment with hydrating and keratolytic agents.

Only a new frameshift small deletion, namely, c.76_85del (p.Thr26Serfs*), was identified by NGS within the first coding exon (exon 3) of the *CYP4F22* gene, apparently in homozygous state, as also confirmed by Sanger sequencing (Figure 4B).

As we could not perform segregation analysis, we evaluated the possible presence of an overlapping exon deletion [25,27,28]. Quantitative analysis of the read depth was performed for each exon of *CYP4F22* in the patient in comparison to three controls, giving a relative ratio consistent with the presence of a heterozygous deletion removing the whole exon 3; the remaining exons were in double dose (Figure 4B). The result was confirmed by real-time qPCR (Figure 4C). Thus, patient 4 actually resulted to be a compound heterozygous for two novel deleterious deletions within *CYP4F22.* No other variants were identified in the remaining ARCI-associated genes analyzed.

## 4. Discussion

Since the year 2000, a genetic test looking for *TGM1* mutation is being applied to ARCI patients referred to our center, which achieves about 40% detection rate in our population [9,11,29,30]. A number of patients that remained undiagnosed contacted us periodically for having information about any technological advances that could unravel their condition.

Among them there were the four patients reported here. Notably, they were affected by CIE of variable severities (Table 3).

The CIE phenotype is characterized by diffused dry scaling with underlying erythema. Scales are usually fine and white all over the body, whereas they are brownish thick and plate-like on the lower limbs [1,2,3,4]. Collodion membrane at birth is frequently present. Ectropion and eclabium are infrequent. Also reported are hypoplasia of nasal and auricular cartilages, decreased sweating with heat intolerance, palmoplantar hyperkeratosis, and nail dystrophy [10].

CIE has been associated with pathogenic variants in other ARCI genes besides *TGM1*, including *ABCA12*, *ALOX12B*, *ALOX3*, *NIPAL4*, *CYP4F22*, and *PNPLA1* [1,2,3]. Therefore, to identify the molecular cause of ichthyosis in our patients, we used a panel-based NGS assay that targeted the coding regions of most genes implicated in Mendelian disorders, including ARCI. This approach can be applied to genetic testing of patients affected by the most disparate inherited diseases to discover the causative gene and variants associated with a specific phenotype [17,18,19]. Molecular data we have obtained are very encouraging, since likely pathogenic variants have been identified in all the patients. Although such a panel targets a very large number of genes, coverage of exons and flanking splice sites of the 13 ARCI genes is 100%, with minimum read depth of 15X; 99.98% of targets has at least 20X. Importantly, read depth has been sufficient to successfully reveal a heterozygous deletion involving exon 3 of the *CYP4F22* gene. We discovered this deletion by formulating a calculation method for quantitative analysis of read depth in the genes under study, which has proven to be sensitive enough to detect heterozygous deletions. This method worked well with ARCI and with other genes (data not shown), thereby indicating it is virtually applicable to any gene targeted in the panel.

Except for one, the variants we identified in our patients have not been previously associated with ARCI. According to other studies, mutation of *ALOX12B* is the primary cause of CIE in our Italian patients [26]. Indeed, in three patients we identified likely pathogenic variants, including a novel pathogenic frameshift, a known missense, and a large deletion, a type of mutation never described before in *ALOX12B*. Two additional missense variants were classified as VUS by Varsome because they were not associated previously with the disease; however, according to ACMG criteria, the fact that they segregated in association with a pathogenic allele in affected patients increased their relative pathogenicity score [24].

As summarized in Table 3, severity of the CIE phenotype was quite variable among our patients. Interestingly, among the *ALOX12B*-mutated patients, the phenotype of patient 3 was very mild and therefore can be considered a case of SICI [31]. SICI due to *ALOX12B* has been often associated with missense variants [31]. To explain the relevant epidermal improvement observed after birth, it has been proposed that the resulting amino acid changes negatively affect arachidonate 12-lipoxygenase (12R-LOX) activity only under in utero conditions as a consequence of transient protein misfolding [31]. This model cannot be applied to our patient 3, which is homozygous for a large deletion that removes *ALOX12B* sequences encoding the whole catalytic domain of the 12R-LOX enzyme. As this genomic variation leads to a null-allele, we speculate that 12R-LOX deficiency alone is necessary, but not sufficient to hold the ichthyosis phenotype severe after birth.

This hypothesis is in agreement with the genotype-phenotype relationship in patients 1 and 2, who also have *ALOX12B* mutations. Indeed, patient 1 showed the most severe CIE phenotype and had a frameshift variant associated in trans with a deletion that removes the trinucleotide encoding Tyr687, located within the catalytic domain of the 12R-LOX enzyme. Notably, this patient was also compound heterozygous for missense variants in *ABCA12*; homozygous missense variants in *ABCA12* have been associated with LI and CIE [3,32,33].

The 12R-LOX enzyme oxidates linoleic acid to ceramide; the *ABCA12* gene product is a transmembrane transporter loading glucosylceramide into lamellar bodies (LBs). Both of these proteins participate in the epidermal lipid synthetic pathway, leading to the formation of corneocyte lipid envelope (CLE) and extracellular lamellar membranes [34,35]. Notably, deficiency of 12R-LOX affects CLE formation, whereas loss of the ABCA12 transporter impairs LBs production in knockout mice [34]. In this context, the complex genotype of patient 1 could explain his severe CIE phenotype; indeed, besides the 12R-LOX deficiency caused by the two pathogenic variants p.Leu451Alafs* and p.Tyr687del, the *ABCA12* missense variant p.Glu2138Ala can be reasonably considered a hypomorphic allele that negatively modified the patient’s phenotype [19,33]. Interestingly, patient 1 was born preterm as collodion baby, and premature birth is a clinical feature associated with *ABCA12* mutations; moreover, he has overfolded ears, which were noted in many patients with *ALOX12B* mutations [10].

Similarly, the CIE phenotype of patient 2 was slightly more severe than that of patient 3, although her *ALOX12B* alleles encode for missense variants, namely p.Ser16Leu and p.His398Tyr. In particular, and similarly to patient 1, patient 2 had marked palmoplantar keratosis, which is a clinical sign of disease severity not often observed in *ALOX12B*-related patients [10]. Interestingly, she was also heterozygous for a rare missense variant in *SULT2B1*. This gene encodes the cholesterol sulfotransferase SULT2B1 involved in the regulation of epidermal cholesterol metabolism; its deficiency causes a cholesterol sulfate decrease and cholesterol accumulation, and results in an ARCI type with clinical signs overlapping with LI and CIE [36]. Deficiency of SULT2B1 affects cholesterol synthesis, which is essential to form LBs; notably, either the deficiency or accumulation of cholesterol may disturb the proper formation and processing of LBs during the transition from keratinocyte to corneocyte [36]. Therefore, we can reasonably consider the rare *SULT2B1* variant p.Ser16Leu, although classified as likely benign by Varsome, a hypomorphic allele that worsens the patient’s skin structure already impaired by 12R-LOX deficiency.

Lastly, patient 4 was a compound heterozygote for two different deleterious deletions in the *CYP4F22* gene (Table 2 and Table 3). One is a new large deletion removing exon 3, which contains the ATG translation start site. *CYP4F22* encodes fatty acid ω-hydroxylase essential for acylceramide synthesis and therefore for CLE formation [37], and mutations in this gene have been identified in LI, CIE, and SICI patients [38,39]. It should be noted that patient 4, who was born with collodion membrane, has a quite severe phenotype, with particularly serious hyperkeratosis of the elbows and knees (Table 3). Nevertheless, the evident and diffuse erythroderma makes this phenotype similar with the CIE phenotype of the other patients described herein. Moreover, the phenotype of patient 4, despite complete enzyme deficiency consequent to the presence of two *CYP4F22* pathogenic null alleles, was similar with phenotypes previously associated with missense pathogenic variants in this gene, thereby supporting the absence of an association between mutation type and ichthyosis severity [38].

In summary, our study demonstrated that multi-gene NGS in clinical settings is a powerful and sensitive technology for the diagnosis of very rare heterogeneous genetic disorders, such as ARCI. This methodology successfully achieved molecular diagnosis in all four analyzed ARCI patients by identifying seven pathogenic variants in *ALOX12B* and *CYP4F22*. The multi-gene analysis allowed the identification of additional heterozygous variants in different ARCI genes, i.e., *ABCA12* and *STUB2B1*, which could be negative modifiers of the CIE phenotype associated with *ALOX12B* deficiency, in our patients. Among the four novel variants, there were two large deletions removing crucial sequences of *ALOX12B* and *CYP4F2*, a mutation type never identified before in these genes. Differently from most *TGM1*-positive LI Italian patients, three out of four of our CIE patients had nonconsanguineous parents and are compound heterozygotes, which indicates this condition is more frequent than expected [6]. Likely, most patients remain clinically undiagnosed because they are affected by the mildest forms of CIE or SICI that overlap with the ichthyosis vulgaris phenotype, especially in adults [40].

Our analysis, although limited to four ARCI patients, supports previous evidence that *ALOX12B* is the main gene associated with CIE and enlarges the number of pathogenic alleles currently listed in public or disease-specific databases, including also the known variants herein associated with the disease for the first time. Similar to other genes associated with inherited diseases [25,41], large deletions have been found among the *ALOX12B* and *CYP4F22* disease alleles. Therefore, we alert on the significant possibility that the presence of this type of CNVs can cause molecular diagnostic pitfalls [27,28].

All the sequence variants identified in our CIE patients affect ARCI genes involved in the skin pathways of lipid metabolism or lipid transport that lastly lead to the building of the CLE, which is essential for proper function of epidermal barrier. In agreement, the phenotypes of our four patients displayed many similarities, but at the same time, they showed significant differences that cannot be explained by looking exclusively at the defect of a single gene. Changing perspective, looking at the genotypes, our data gave hints that other genetic determinants could contribute to the clinical variability of ARCI. In this context, molecular methodologies that screen multiple genes, besides early detection and/or differential diagnosis of rare inherited diseases, can answer unsolved questions concerning clinical variability of the same monogenic disorder [42,43].

## Figures and Tables

**Table 1:** List of primer pairs used for qPCR gene dosage assay.

Gene	Exon	Sequence	Tm (°C)	Fragment Size
*ALOX12B*	1	5′-CATCCACGGCATCTTCTATC-3′	60.5	200 bp
	1	5′-AGAGATCTGGGACATGGGCG-3′	67.1	
	9	5′-CCTTCTCATACTCCCTTCTG-3′	56.3	360 bp
	9	5′-GAAGTCCCTATGCCAAGCCC-3′	65.3	
	10	5′-CTTCAGCCCTCTCTCTTCAT-3′	58.5	449 bp
	10	5′-TCCTCCTCTTCATCTAACTG-3′	54.3	
	15	5′-GGGATGGGGGAGGATAACTA-3′	62.2	745 bp
	15	5′-AGAATGGGGAGAGGAGAGAC-3′	59.6	
*CYP4F22*	3	5′-TGTGCTGGGAACCTTCTGTG-3′	64.7	383 bp
	3	5′-CCTACCTATTACCCTGCACA-3′	57.5	

**Table 2:** List of autosomal recessive congenital ichthyoses (ARCI)-related genomic variants identified in our Italian patient with the congenital ichthyosis erythroderma (CIE) phenotype.

ID	Gene	Nucleotide Change	Protein Effect	RefSNP Database	Variant Significance
P1	*ABCA12*	c.6413A>C (het)	p.Glu2138Ala	--	VUS
	*ABCA12*	c.7093G>A (het)	p.Asp2365Asn	rs726070 MAF = 0.028	benign
	*ALOX12B*	c.1350dupG (het)	p.Leu451Alafs*	--	pathogenic
	*ALOX12B*	c.2065_2067del (het)	p.Tyr687del	rs1414018786 MAF = 0.000008	pathogenic
P2	*ALOX12B*	c.47C>T (het)	p.Ser16Leu	rs147784568 MAF = 0.000012	likely pathogenic
	*ALOX12B*	c.1192C>T (het)	p.His398Tyr	rs752176414 MAF = 0.000016	likely pathogenic
	*SULT2B1*	c.912G>A (het)	p.Met304Ile	rs142168444 MAF = 0.001	likely benign
P3	*ALOX12B*	delExon3_15 (homo)	null	--	pathogenic
P4	*CYP4F22*	c.76_85del (het)	p.Thr26Serfs*	--	pathogenic
	*CYP4F22*	delExon3 (het)	null	--	pathogenic

CIE: congenital ichthyosis erythroderma; ID, patient identification code; het, heterozygous; homo, homozygous; MAF, minor allele frequency; VUS, variant of uncertain significance. Mutation numbering is based on the NCBI reference sequences of *ABCA12* (NM_173076.3; NP_775099.2), *ALOX12B* (NM_001139.3; NP_001130.1), *CYP4F22* (NM_173483.4; NP_775754.2), *SULT2B1* (NM_177973.2; NP_814444.1). For cDNA numbering, +1 corresponds to the A of the ATG translation initiation codon, which is codon 1.

**Table 3:** Clinical features and associated genotypes of CIE patients under study.

ID	Gene	Variants	Sex/Age	Phenotype	Additional Signs
P1	*ABCA12*	c.6413A>C	M 32	CB, diffused white-gray scaling and erythema, hyperkeratosis on legs, arms and scalp, PPH	Premature birth, deformed auricles, anhidrosis, burning sensation, conductive hearing loss
	*ALOX12B*	c.1350dupG/2065_2067del			
P2	*ALOX12B*	c.47C>T/1192C>T	F 40	CB, fine white scaling and mild erythema, mild keratosis on legs and arms, PPH	Hypohidrosis, skin erythema in a hot environment, frequent itching
	*SULT2B1*	c.912G>A			
P3	*ALOX12B*	exon3_15del/exon3_15del	M 35	CB; mild face erythema, palmar hyperlinearity	None
P4	*CYP4F22*	c.76_85del/exon3del	F 43	CB, diffused white-gray scaling and erythema, hyperkeratosis on legs and arms, PPH	Brownish scales on trunk and back, hypohidrosis, hyperkeratosis of elbows and knees

CB, collodion baby; CIE, congenital ichthyosis erythroderma; ID, patient identification code; F, female; M, male; PPH, palmoplantar hyperkeratosis.
